# Evaluating urban surface water quality in Luton

**DOI:** 10.1007/s10661-019-7264-z

**Published:** 2019-02-09

**Authors:** Tahmina Ajmal, Tochukwu Kene Anyachebelu, Marc Conrad, David M. Rawson

**Affiliations:** Department of Computer Science and Technology, University of Bedfordshire, Luton, LU1 3JU UK

**Keywords:** Surface water, Water quality index, Environmental assessment, River Lea

## Abstract

Using a single numerical value to indicate the quality of water, a so-called Water Quality Index (WQI) is a well-established way of rating the overall water quality status of a given water body. During the last few years, researchers in the water sector have developed different such indices to address their specific needs. In this study, we attempt to obtain a WQI formula suited for evaluating the water quality of the River Lea. We have selected four different sites on the River Lea and explore the possibility of monitoring using a minimum number of parameters only. The results obtained are very encouraging and provide a strong indication that only three parameters are enough to indicate water quality of a water body.

## Introduction

A water quality index (WQI) is a very useful tool that allows water researchers to get quantitative indication of water quality. The WQI is obtained by calculating the score for each measured parameter and combining them into one overall score. This index can be, however, calculated in different ways (Sutadian et al. 2016, Lumb et al. 2011). The selected calculation method is based on various factors like the governing environmental body, parameters measured, parameters of importance etc. We observe therefore that while calculating the WQI as a strategy has been widely applied, there is no standardised formula: the calculation varies, depending on the local conditions (Alobaidy et al. 2010, Akkaraboyina and Raju 2012, Anyachebelu et al. 2015, Debels et al. 2005).

One of the most generalised methods is the Objective Water Quality Index method adapted by Bascaro’n (1979). This is given by Eq. (1):

1 $$ {WQI}_{obj}=k\frac{\sum_{i=1}^n{C}_i{P}_i}{\sum_{i=1}^n{P}_i} $$

Here, *C*_*i*_ = value of the corresponding parameter after normalisation; *P*_*i*_ = parameter weight (this is given as values 1–4 with 4 being assigned to the most important parameters with regard to aquatic life protection); *n* = total number of parameters monitored; *k* = constant value based on visual assessment of the water body.

The parameters for the calculation of the WQI can then be selected based on their importance in indicating water quality within a given context. Among the strong indicators are dissolved oxygen, seen as a highly important parameter, assesses the quality of water for aquatic life; conductivity or total dissolved solids (TDS) shows that there are salts, mineral acids, similar contaminants present in a water body; turbidity is linked with cases of suspended materials in water as well as the bacteriological contamination.

Pesce and Wunderlin [2000] propose the concept of a minimum WQI that is based on normalised values of three measured parameters. This is given in Eq. (2)

2 $$ {WQI}_{\mathrm{min}}=\frac{C_{\mathrm{Dissolved}\ \mathrm{oxygen}}+{C}_{\mathrm{Turbidity}}+{C}_{\mathrm{Conductivity}\ \mathrm{or}\ \mathrm{TDS}}}{3} $$

Conesa Fernandes-Vitora (1995) curves are used to normalise the different parameters in the same unit. The weight values and normalisation factors are based on expert judgement and a series of literature to incline with the local conditions (Dos Santos Simoes et al. 2008).

In this work, we present the analysis of data from a number of different sites on the river Lea in Luton in an attempt to obtain a suitable WQI calculation method for Luton Lea.

## Study design

In this study, we are focussing on the river Lea near Luton that forms an essential part of the historic River Lea that joins the River Thames near Bow. The River Lea originates from a natural spring at Leagrave (north of Luton) and includes Houghton Brook, Lewsey Brook and Carbrook as its tributaries. The geology of the study area consists of Chalk (from surrounding Chilterns) within the Lambeth Group along the northeast and southwest sides of Luton and Glaciofluvial deposits along the river. This underlying geology creates a highly variable environment for possible runoff infiltration and related groundwater flooding. The river Lea flows through Bedfordshire, Hertfordshire and Greater London. The surrounding area is mainly urban, and the water quality is affected by different factors including navigation, abstraction, misconnections and water runoff from roads and nearby areas. In particular, the dissolved oxygen level is almost persistently low across the whole of the Lea (Patroncini et al. 2014). Additional urban growth in the Luton area has put in cumulative pressures on water resources in the area, and as a result the water quality in the area has a poor ecological and chemical status (Environment Agency 2015).

**Fig. 1:**
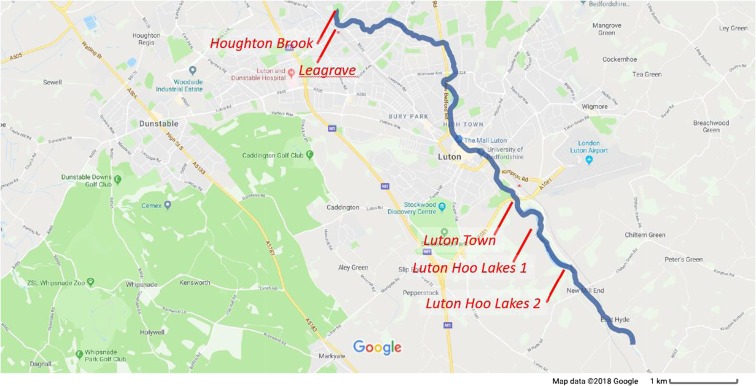
Map of Luton showing the river Lea (blue) and the location of the five monitoring sites (red)

The sites selected for this work are at Houghton Brook, Leagrave, Luton Town, and two sites on Luton Hoo Lake. These sites are indicated in the map in Fig. 1.The measurements from Luton Hoo Lake are from two continuous monitoring stations that are maintained by the Environment Agency and Luton Airport respectively. All the other three sites were manually sampled, and data is provided by the Environment Agency. Manual sampling, where available, is done once every 2 weeks and encompasses a larger range of physical and chemical parameters. The two continuous monitoring sites use six common parameters—pH, dissolved oxygen, ammonium, conductivity, turbidity and temperature.

The data obtained from these sites along Luton Lea is evaluated here to calculate its WQI. This is done in at least two ways for each site in order to determine the possibility of getting a minimum parameter index value that reflects the same or similar value as expressed by using more physiochemical parameters for the calculation. That is, an attempt is made to evaluate the possibility of minimum monitoring. For the first calculation, the full WQI is used—based on all the parameter data available for the corresponding site. For the second calculation, the minimum WQI is used as described by Eq. (2). The data is evaluated over at least one calendar year to get a fair assessment. In some cases, where earlier data was available assessment has been extended to two calendar years.

**Fig. 2:**
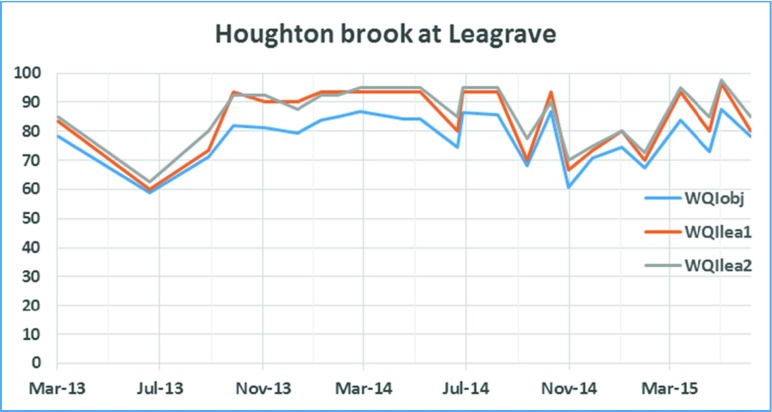
WQI for Houghton brook at Leagrave

**Fig. 3:**
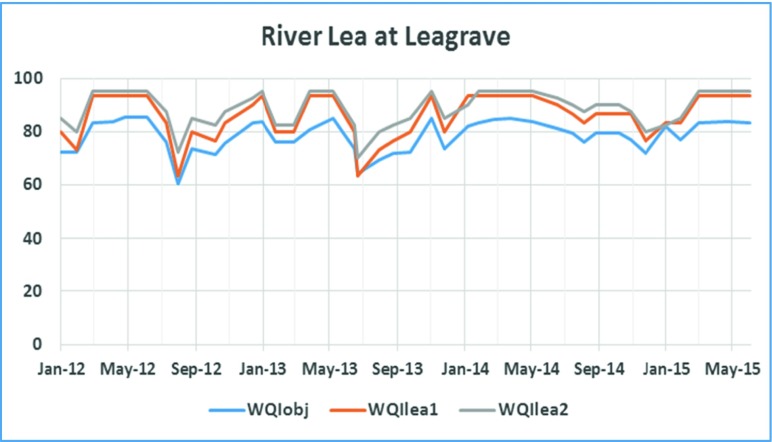
WQI calculation plot for River Lea at Leagrave

For the two sites in Leagrave, Houghton brook (results in Fig. 2) and River Lea (this site is labelled as Leagrave in Fig. 1; results shown in Fig. 3), WQIobj is evaluated as in Eq. (1) using the following parameters: pH, temperature, conductivity ammonia, nitrate, nitrites, orthophosphate and dissolved oxygen.

To obtain a minimum evaluation index for these sites, WQI_lea1_ and WQI_lea2_ were derived from Eq. (2). These are shown in Eqs. (3) and (4) below:

3 $$ {WQI}_{lea_1}=\frac{C_{\mathrm{Conductivity}}+{C}_{\mathrm{Dissolved}\ \mathrm{oxygen}}+{C}_{\mathrm{Ammonia}}}{3} $$

4 $$ {WQI}_{lea_2}=\frac{C_{\mathrm{Conductivity}}+{C}_{\mathrm{Dissolved}\ \mathrm{oxygen}}+{C}_{\mathrm{Phosphate}}+{C}_{\mathrm{Ammonia}}}{4} $$

For the third manual sampling site at River Lea above Luton Hoo (Fig. 3), WQI_*obj*_ is evaluated using the following parameters: pH, temperature, ammonia, nitrate, nitrite, orthophosphate and dissolved oxygen. The WQI_lea3_ is calculated using the normalised values for ammonia, phosphate and dissolved oxygen as given in Eq. 5 below:

5 $$ {WQI}_{lea_3}=\frac{C_{\mathrm{Ammonia}}+{C}_{\mathrm{Phosphate}}+{C}_{\mathrm{Dissolved}\ \mathrm{oxygen}}}{3} $$

**Fig. 4:**
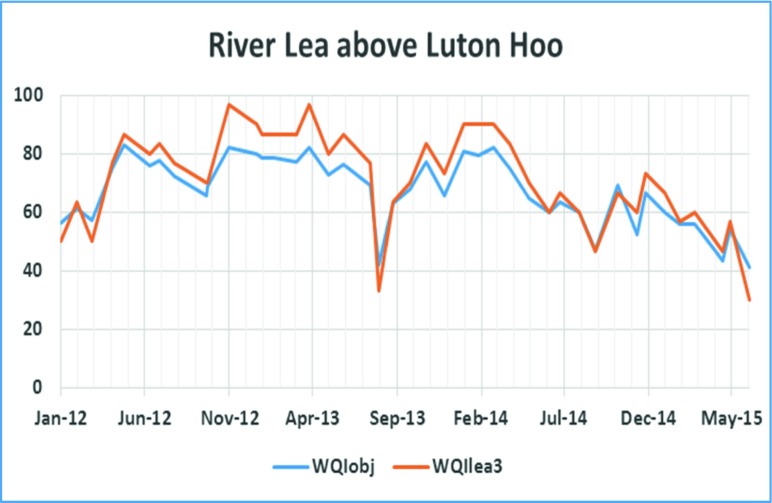
WQI for River Lea above Luton Hoo

**Fig. 5:**
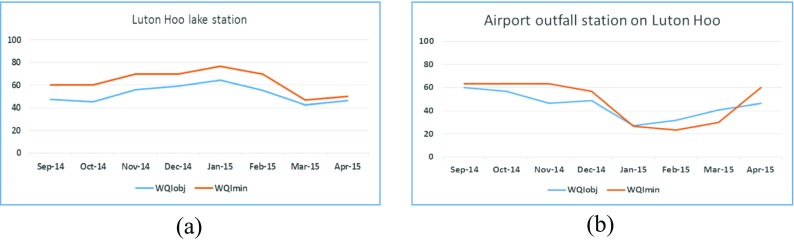
WQI calculations **a** for Luton Hoo Lake Station1 maintained by Environment Agency and **b** for Luton Hoo Lake Station 2 maintained by Luton Airport

For the two sites on Luton Hoo Lake with continuous monitoring, measured parameters include pH, dissolved oxygen, ammonia, conductivity, turbidity and temperature. So, WQI_*obj*_ is calculated for a monthly average using all these parameters while WQI_*min*_ is calculated using conductivity, dissolved oxygen and turbidity only. These results are shown in Figs. 5 (a) and (b) below. This data is collected every 15 min. The water quality index evaluated for these two sites at Luton Hoo Lake is hence calculated using averaged values. As a result, they seem less discrete in comparison to Figs. 3 and 4.

## Discussion on results

All the results shown in Figs. 2, 3, 4 and 5 indicate a fair agreement in the results of the two methods used. The minimum method, although following the same contours as the objective method, gives a more optimistic value. Water quality can be rated (Tyagi et al. 2013) based on WQI values as ‘excellent’ (90–100), good (70–90), medium (50–70), bad (25–50) and very bad (less than 25). From our results, we note that the ‘bad’ WQI period is usually the summer peak period and could be explained by the high temperatures adversely affecting the amount of dissolved oxygen available in the surface water. In addition, at least sometimes, this could also be attributed to less water, indicating a concentration of contaminants.

It is observed that Luton Hoo lake was moderately polluted for most of the observed period with some cases of excess pollution recorded in September to October 2014 and March to April 2015. The airport outfall station on Luton Hoo exhibits excess pollution for most of the period from November 2014 to April 2015 with the worst situations occurring around February 2015. This could be attributed to the use of anti-freeze by the airlines during the winter months. Within the 27 months period for which the lake has been monitored, the different parameters measured at the various stations across the River Lea and Luton Hoo Lake have helped in assessing the water quality of the water bodies for aquatic life habitation.

Exploring the parameters individually shows that pH achieved a normalisation of 80% and above for all the stations which was within the recommended range for surface waters used for aquaculture purposes. All other parameters fluctuated in different ranges across different stations. This is one of the major reasons for exploring the use of water quality index and its evaluation.

**Fig. 6:**
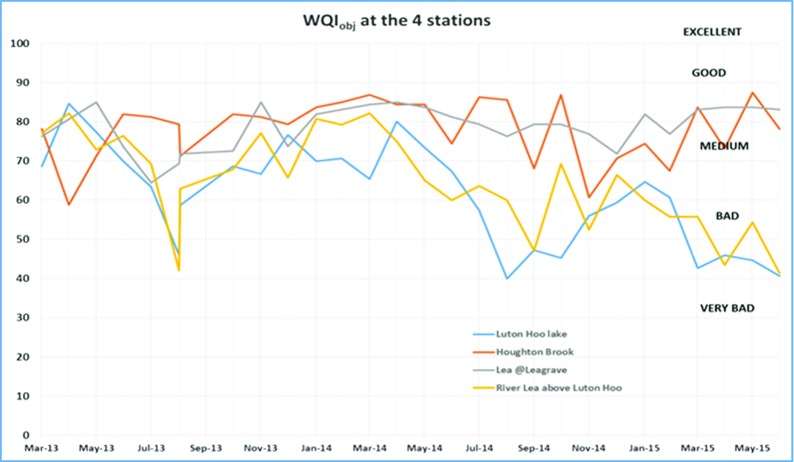
WQIobj calculation at 4 stations over two annual cycles

**Fig. 7:**
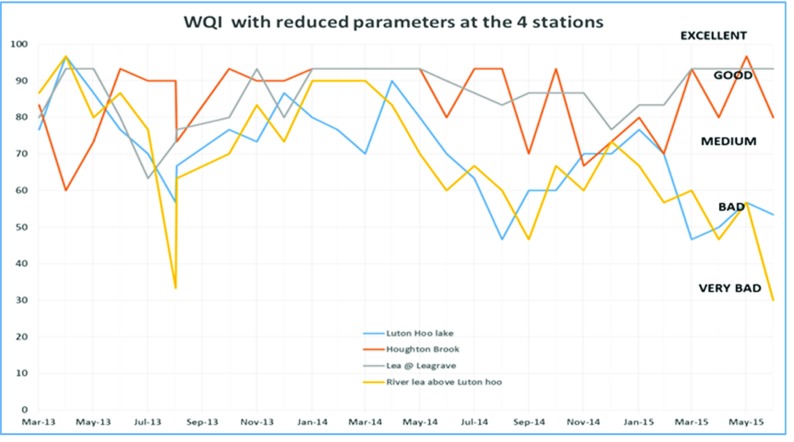
WQImin calculation with reduced parameters at the 4 stations over the duration of two annual cycles

Data from all the monitoring stations is combined in Figs. 6 and 7. Luton Hoo airport outfall data is discounted here because this data became available only from Sept 2014 when this monitoring station was re-installed. It is observed that the WQI value for Luton Hoo station and River Lea above Luton went as low as 40 in August 2013, August 2014 and June 2015. The same trend is obvious in both the graphs. Data from the two stations Houghton brook and River Lea at Leagrave remain mostly within the ‘medium’ quality to ‘good’ quality indicating slight pollution at these stations. This could be because both of these monitoring points are located upstream so there is a lower probability of contamination from human sources.

The WQI with reduced parameters as derived in Eqs. (2),(3),(4) and (5) utilised fewer parameters to determine the water quality index in a less expensive manner. In examining the WQI chart in Fig. 7, there is an indication of the WQI value falling within the ‘bad’ quality level in August 2013, August 2014 and June 2015 which gives the same trend as exhibited by the WQI which was carried out using more parameters (Fig. 6). A closer look at the Luton Hoo Lake with the parameters measured remotely online and the River Lea above Luton Hoo with its parameters measured manually shows a similar trend with WQI evaluated. There is a conviction that values obtained at these points reflect the quality of the water irrespective of the method of sampling.

## Conclusion

The minimal index with three parameters as defined in this work has been found sufficient to evaluate the surface water quality of the River Lea with respect to aquatic living. This was compared with the objective WQI which makes use of more parameters for evaluation and exhibits a similar trend. The minimal WQI was explored both with manual monthly values from monitoring stations and online 15-min interval monitoring stations. We found that the trends were similar but with slightly different index values. We see from the trend analysis of the various stations that the Houghton brook and River Lea at Leagrave stations exhibited better water quality status than the River Lea above Luton Hoo and Luton Hoo lake stations. This is attributed to the point source contaminants that get into the river as it moves from Leagrave downstream into Luton Hoo passing through Luton.

Results are compared in Figs. 2, 3, 4, 5, 6 and 7 using both methods and although parameters used to calculate WQI_obj_ are different, it shows a similar trend with WQI_min_. The parameters used for the evaluation of WQI_min_ are selected based on the importance for aquatic life and there is some flexibility in the choice of parameters. This makes the index highly applicable in varied scenarios with limited resources for monitoring in place. In most cases, both methods indicate a similar trend in the water quality. It is interesting to note that in cases of sudden changes, WQI_min_ is either matched with WQI_obj_ or it gives a lower value. This is especially useful for an early warning system. Thus, the minimal water quality index, WQI_min_, is a valuable tool for decision makers in water quality monitoring as it gives reasonable results at a lower cost for a given monitoring program.
